# Dynamics of Gut Microbiota Diversity During the Early Development of an Avian Host: Evidence From a Cross-Foster Experiment

**DOI:** 10.3389/fmicb.2018.01524

**Published:** 2018-07-09

**Authors:** Aimeric Teyssier, Luc Lens, Erik Matthysen, Joël White

**Affiliations:** ^1^Terrestrial Ecology Unit, Department of Biology, Ghent University, Ghent, Belgium; ^2^Evolutionary Ecology Group, Department of Biology, University of Antwerp, Antwerp, Belgium; ^3^Laboratoire Evolution et Diversité Biologique, UMR 5174 Centre National de la Recherche Scientifique–Université Paul Sabatier–Institut de Recherche pour le Développement, Toulouse, France

**Keywords:** microbiome, 16S amplicon metabarcoding, ontogeny, colonization, cloaca, songbird, growth, bacterial communities

## Abstract

Despite the increasing knowledge on the processes involved in the acquisition and development of the gut microbiota in model organisms, the factors influencing early microbiota successions in natural populations remain poorly understood. In particular, little is known on the role of the rearing environment in the establishment of the gut microbiota in wild birds. Here, we examined the influence of the nesting environment on the gut microbiota of Great tits (*Parus major*) by performing a partial cross-fostering experiment during the intermediate stage of nestling development. We found that the cloacal microbiota of great tit nestlings underwent substantial changes between 8 and 15 days of age, with a strong decrease in diversity, an increase in the relative abundance of Firmicutes and a shift in the functional features of the community. Second, the nesting environment significantly influenced community composition, with a divergence among separated true siblings and a convergence among foster siblings. Third, larger shifts in both microbiota diversity and composition correlated with lower nestling body condition. Our results shed new light on the dynamics of microbial diversity during the ontogeny of avian hosts, indicating that the nest environment continues to shape the gut microbiota during the later stages of nestling development and that the increase in gut diversity between hatching and adulthood may not be as linear as previously suspected. Lastly, the microbiota changes incurred during this period may have implications for nestling body condition which can lead to long-term consequences for host fitness.

## Introduction

The digestive tract of all vertebrates harbor microbial communities comprised of diverse bacterial taxa varying in abundance and functional traits (Zilber-Rosenberg and Rosenberg, 2008; McFall-Ngai et al., 2013). These gut microbiota play a fundamental role for host health and fitness, as they mediate processes such as digestion and nutrient synthesis (Cummings and Macfarlane, 1997), immunomodulation (Round and Mazmanian, 2009), and pathogen defense (Fukuda et al., 2011) and more widely influence life-history traits (Sison-Mangus et al., 2015) or even behavior (Ezenwa et al., 2012).

In vertebrates, the gut microbiota is shaped by both external factors, such as diet and host environment (Flint et al., 2015; Teyssier et al., 2018a), which influence the pool of potential colonists of the gut, and by host-associated factors, such as genotype, sex or immune status (Bevins and Salzman, 2011), which impose selective filters on community composition. Bacterial communities remain relatively stable in adult organisms, whereas they are much more transient and dynamic in juveniles. In mammals, which comprise the most studied taxa, the gut harbors very few bacteria at birth and is rapidly colonized by several microbial taxa shortly after (Koenig et al., 2011). During the first weeks following birth, gut bacterial diversity, and abundance of the newborn remain low. In humans, bacterial diversity increases with age at an exponential scale within the first 3 years and continues to increase until adulthood, although at a lower rate (Kostic et al., 2013). In terms of taxonomic composition, the microbiota undergoes large fluctuations during the first months/years of life, especially when the diet changes from maternal milk to solid food (Laforest-Lapointe and Arrieta, 2017). After the first years of life, the maturation of the microbiota leads to an increase in stability, with adults hosting a well-established microbiota that is less sensitive to modifications (Kostic et al., 2013).

In birds, the mechanisms of microbiota acquisition and development are likely to differ somewhat. While mammal guts are initially colonized by vaginal (and possibly placental, see Carmen Collado et al., 2016) maternal bacteria and then via lactation, bird embryos develop in a closed and essentially sterile environment, which is the egg. Hence, the first external bacteria to colonize the gut of birds most likely originate from the nesting environment and then from parental feeding (in altricial species), although the possibility of vertical transmission of bacteria during ovogenesis cannot be ruled out (see Ding et al., 2017 and Grond et al., 2017 for contrasting results). The gut microbiota of birds subsequently undergoes a series of community successions which are thought to be largely influenced by environmental factors and more specifically the environment in which the chicks are reared (Hird et al., 2014). In this context, studies in natural bird populations may be more relevant for understanding the factors mediating early microbiota development than poultry studies (Amato, 2013; Hird, 2017).

Although still scarce, several recent studies have investigated the early establishment of the gut microbiota in natural populations of wild birds, such as in seabirds (Barbosa et al., 2016; Dewar et al., 2017), shorebirds (Grond et al., 2017), and passerines (*Hirundo rustica*; Kreisinger et al., 2015). These studies collectively show that gut communities undergo an increase in diversity in the first stages of nestling development accompanied by strong fluctuations in community composition. As chicks grow older, gut communities converge toward more stable communities composed of less transient bacterial taxa (see for e.g., van Dongen et al., 2013; Grond et al., 2017). However, the factors influencing such early microbiota successions remain poorly understood in the wild, in particular the role of the rearing environment. Pioneering work by Lucas and Heeb (2005) in blue tit (*Cyanistes caeruleus*) and great tit (*Parus major*) nestlings revealed through cross-fostering that environmental factors were more important than host species in determining gut microbial community structure, whereas Ruiz-Rodriguez et al. (2009) showed the opposite in magpie *(Pica pica*) and cuckoo (*Cuculus canorus)* nestlings raised in the same nest.

The goal of this study is to investigate the early development of the gut microbiota communities in wild passerine nestlings. To address this question, we sampled the cloacal microbiota of great tit nestlings before and after a partial cross-fostering experiment that took place during the intermediate stage of nestling development (age 8–15 days). This approach allowed us to examine the influence of the nesting environment (nest and nestmates) on microbiota acquisition at that stage of nestling development, by comparing gut community dynamics in fostered and control nestlings. We were thus able to test different hypotheses regarding early microbiota acquisition dynamics. If the gut microbiota stabilizes in the first days after hatching (Grond et al., 2017) and the early rearing environment is the main factor contributing to microbiota establishment, then we expect little change in gut microbiota composition between 8 and 15 days and little effect of the experimental cross-fostering on microbiota dynamics. On the contrary, if the nesting environment continues to shape the microbiota during later stages of nestling development, we then expect a greater change to occur in fostered nestlings than in control nestlings. We further expect the microbiota of true siblings reared in different nests to grow increasingly dissimilar and that of foster siblings to become more similar with age. Moreover, by comparing nestlings that were fostered themselves or received foster siblings we can disentangle the contribution of nest environment *per se* versus the influence of nestmates. Finally, we provide information on functional changes in microbiota composition, and examined whether changes in gut microbiota are associated with individual condition.

## Materials and Methods

### Study Site

The study was performed during the breeding season 2016 (May–June 2016) in a woodland area called “Boshoek” in northern Belgium (51°08′N, 4°32′E). This area contains woodland patches equipped with standard nest boxes in which great tits breed (Matthysen et al., 2005). Nest boxes are checked repeatedly during the breeding season to determine laying date, clutch size, and breeding success.

### Cross-Fostering Experiment

We performed a partial cross-fostering experiment between great tits nests and sampled nestling microbiota at two different developmental stages: at 8 days old (just before the cross-fostering) and at 15 days old (1 week after the cross-fostering). Cross-fostering was done between two randomly selected nests with at least six nestlings of the same age (8 days old). At day 8 (D8), all nestlings were ringed and six randomly selected nestlings were weighed and had their cloacal microbiota sampled. Three nestlings of each nest were then randomly chosen and placed in the other nest. At day 15 (D15), all six nestlings were re-sampled for microbiota, weighed, and their tarsus length measured. After sampling, nestlings were returned to their initial nest. We used 26 nests in this experiment, of which 18 nests were cross-fostered and 8 nests were used as control. In the latter, microbiota was also sampled at D8 and D15, but all nestlings remained in their original nest. Since, we had no nestling mortality between D8 and D15, this resulted in a total of 156 nestlings sampled. These belong to two main experimental treatments, control nests and cross-fostered nests (hereafter termed CF), the nestlings of the latter group being further subdivided into two categories, those that remained in their initial nest (CFstay) and those moved to a new nest (CFmove). This study was carried out in accordance with the recommendations of the Guidelines for Animal Care and Treatment of the European Union and the protocol approved by the Flemish Ministry for Environment (license number: ANB/BL/FF-V16-00074).

### Gut Microbiota Sampling

We sampled gastrointestinal bacterial communities by sampling the cloaca of nestlings. Cloacal sampling comprises a reliable non-invasive technique to study inter-individual variability in communities present in the gastrointestinal tract and has been successfully used in a number of studies (White et al., 2010; van Dongen et al., 2013; Teyssier et al., 2018a). While each part of the digestive tract harbors specific bacterial communities, there is evidence in birds, that microbial shifts incurred in the higher intestine lead to concurrent shifts in cloacal communities (Newbold et al., 2015). Cloacal bacteria were sampled by gently inserting a sterile pipette tip into the cloaca, injecting 200 μL of sterile phosphate buffered saline then drawing it out again. Samples were immediately placed in sterile vials, kept in a coolbox in the field and later stored at -20°C. Prior to sampling, the exterior of the cloaca was cleaned with alcohol to avoid contamination from bacteria outside the cloaca. At each capture site, we also collected control samples by pipetting 200 μL of the saline solution and waiting for few seconds before putting it back into a sterile vial to check for possible contamination of the pipette tips and the saline solution during sampling and preparation.

### PCR Amplification and High-Throughput Sequencing

Bacterial DNA was extracted using the Qiagen DNeasy^®^ Blood and Tissue Kit and the standard protocol designed for purification of total DNA from Gram-positive bacteria (Qiagen, Venlo, Netherlands). The V5–V6 region of the bacteria 16S rRNA gene was amplified by PCR using the following universal primers: BACTB-F: 5′-GGATTAGATACCCTGGTAGT-3′ and BACTB-R: 5′-CACGACACGAGCTGACG-3′ (Fliegerova et al., 2014). To discriminate samples after sequencing, both forward and reverse primers were labeled at the 5′ end with a combination of two different 8 bp tags. The PCR amplification was performed in a 25 μL mixture containing 3 μL of 1/10 diluted DNA extract, 0.4 μM of each primers, 1 U of AmpliTaq Gold DNA Polymerase (Applied Biosystems, Foster City, CA, United States), 1× of Taq Buffer, 0.24 μL of bovine saline albumin (Promega Corporation, Madison, WI, United States), 0.2 mM of each dNTP, 2.5 mM MgCl_2_, and 12.06 μL water and following this program: initial denaturation at 95°C for 10 min, 35 cycles of denaturation at 95°C for 30 s, hybridation at 57°C for 30 s, and elongation at 72°C for 30 s. All this lab work was done under sterile condition under laminar flux, all materials cleaned with ethanol and sterilized by UV light for 30 min. To avoid PCR bias, all the biological samples were replicated twice. In addition to biological samples, we also used negative and positive controls to check for the PCR effectiveness. Finally, to deal with mistagging, we followed the protocol proposed by Esling et al. (2015) using blank samples (several tag combinations not associated with biological samples). PCR products were tested on electrophoresis gel and then purified and pooled at an equimolar concentration (1 μg of equimolar amplicon pool). The library construction (kit Illumina Biooscientific PCR free) and the sequencing (Illumina MiSeq 250 bp paired-end v3 chemistry) were performed by a biotechnology company (Fasteris SA, Geneva, Switzerland).

### Bioinformatic Analyses

Illumina sequencing data were processed and filtered using the OBITools package (Boyer et al., 2016). First, we aligned paired-end reads in consensus sequences by taking into account the reads’ overlapping quality and kept consensus reads with overlapping quality higher than 50. Second, we assigned reads to their respective sample by allowing zero error in tags and a maximum of two errors on primers. We further excluded reads containing ambiguous bases (other than A, T, G, C) and reads shorter than 100 bp as they are most likely sequencing errors (Bokulich et al., 2013). Remaining reads were then dereplicated and reads that occurred only once in the entire dataset (singleton) were removed. Reads were then clustered into operational taxonomic units (OTUs) based on their similarity calculated from SUMATRA and then clustered with MCL with a threshold of 97% of similarity (following Konstantinidis and Tiedje, 2005). The most abundant sequence of each cluster was considered as the main sequence and the representative sequence for the OTU. The taxonomic affiliation was done by *ecotag* using the SILVA 16S gene data bank (Camacho et al., 2009).

After taxonomic assignation, we obtained 8,979,463 sequences distributed along 7,618 OTUs with on average 10,524 ± 336 sequences by samples. We then applied different filters to this dataset. First, we merged the two replicates of each biological sample by taking the average number of reads for each OTU. In order to account for mistagging (or tag-switching due to PCR chimera, see Esling et al., 2015), the mean abundance of OTUs present in the blanks (tag combinations that were purposefully not used and should not be found) was subtracted from the same OTUs in each sample.

We identified contaminant OTUs (i.e., bacteria that did not come from the biological sample but from extraction or PCR reagents, or technical contamination during lab work) as OTUs with a higher maximum abundance and a higher mean abundance in negative controls than in biological samples. 499 OTUs (11.9% of the initial abundance) were identified as contaminant using these criteria and then removed from the dataset (detail about these OTUs can be found in Supplementary Data Sheet S2). We then removed singleton OTUs and OTUs with a total abundance lower than 0.005% of the dataset’s total abundance (Bokulich et al., 2013). We finally standardized the sequencing depth of each sample by randomly re-sampling 1,000 reads across samples.

Functional characteristics of the bacterial communities were analyzed using PICRUSt (Phylogenetic Investigation of Communities by Reconstruction of Unobserved States, Langille et al., 2013). We first performed closed-reference 97% OTU picking against the Greengenes database (v 13.5), then used the online Galaxy platform^1^ to perform copy number normalization of each OTU, metagenome prediction of each sample and functional predictions categorized into Kyoto Encyclopedia of Genes And Genomes (KEGG) pathways representing gene counts of each predicted metagenome (Kanehisa and Goto, 2000). Metagenome predictions depend on the taxonomic proximity of the bacterial taxa present within the samples to those present in the genome database. The proportion of the sequences that failed to match the Greengenes reference was relatively high with the 97% similarity threshold (25% of the sequences were discarded) so we used a 94% similarity threshold with a better assignation score (only 3.5% of the sequences removed, pattern and info about the discard reads in Supplementary Data Sheet S3).

The average NSTI (Nearest Sequenced Taxon Index) value for the cloacal bacterial communities was 0.063 ± 0.029, which indicates a good coverage (Langille et al., 2013).

### Statistical Analyses

To study the change in cloacal microbiota characteristics with age as well as the effect of the cross-fostering experiment, we first normalized the data by the total abundance within each sample. Microbiota α-diversity was characterized using tree metrics: OTU Richness, which refers to the number of different OTUs present in each sample, the evenness and the Shannon diversity index (H′), which also takes into account the relative abundance of each OTU within samples. OTU Richness was log-transformed to fit a normal distribution and these two diversity indexes were tested with generalized linear mixed effect models. Microbiota characteristics (diversity and taxonomic composition) were analyzed with models containing age and experimental treatment as fixed factors. As the same birds were sampled at D8 and D15, bird and nest identity (at the time of sampling) were both modeled as random effects. A minimal model containing only significant variables was selected through backward elimination of the non-significant variables (R package nlme, Pinheiro et al., 2017). Functional composition was analyzed using the software STAMP (Parks et al., 2014) by comparing mean function abundances with a Welch’s *t*-test with a Benjamin–Hochberg correction.

Microbiota β-diversity was studied using the Jaccard dissimilarity index based on presence–absence community matrices. Microbiota β-diversity was visualized with a non-metric multidimensional scaling (NMDS) and principal coordinates analysis (PcoA) by plotting samples based on their pairwise dissimilarity in a low-dimensional space. We used Mantel tests to investigate correlations between microbiota dissimilarity matrices (Jaccard) at D8 and D15 for the three treatment groups: control, CFstay, and CFmove. We also analyzed the variance partitioning due to environmental factors on dissimilarity matrices using permutational multivariate analysis of variance (ADONIS). ADONIS was done with 1,000 permutations and using the “margin” option in order to test for the marginal effect of each variable whilst accounting for the effect of the other variables of the model. The homogeneity of dispersion among groups (age, nests, and origin of the siblings) was tested using the Betadisper function. The β-diversity analyses were performed with R using the VEGAN package (Oksanen et al., 2007).

Finally, host body condition was estimated using the scaled mass index (SMI), which adjusts the mass of all individuals to that which they would have obtained if they had the same body size, using the equation of the linear regression of log-body mass on log-tarsus length estimated by type-2 (standardized major axis) regression (Peig and Green, 2009). The regression slope of log body mass on log tarsus length was 1.87 and average tarsus length was 19.3 mm. We thus calculated the SMI as body mass × (19.3/tarsus length)^1.87^ (Peig and Green, 2009). We calculated individual weight gain of nestlings by subtracting the weight at D15 with the weight at D8. The correlations between condition and bacterial parameters were tested by models with condition as response variable and microbiota parameters as predictor variables. Only nestlings that gained weight with age were used for the analyses (four individuals removed).

### Data Accessibility

The nucleotide sequences and metadata have been made available through Pangaea (Teyssier et al., 2018b).

## Results

### Changes in Cloacal Microbiota Diversity Indices, Taxonomic, and Functional Composition

**FIGURE 1:**
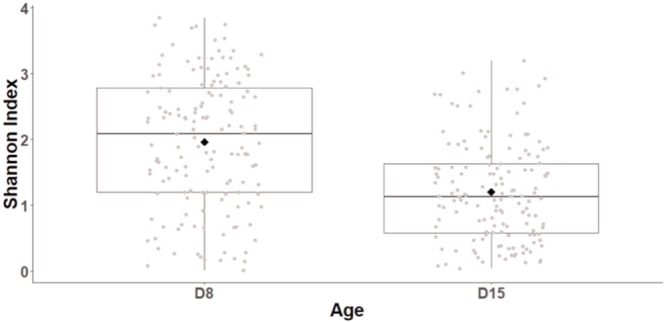
Shannon diversity index of great tits nestling cloacal microbiota at D8 and D15. Median represented by the black line, the mean by the black dot, 25 and 75% quartiles by the lower and upper box and 90% confidence interval by the whiskers.

Age had a major impact of cloacal diversity with significant decreases in OTU richness (LogOTU, GLMM, *F*_1,141_ = 21.9, *p* < 0.0001; D8: 50.51 ± 2.26 OTUs, D15: 36.09 ± 1.92 OTUs), evenness (GLMM, *F*_1,141_ = 6.8, *p* < 0.0001), and Shannon index (GLMM, *F*_1,141_ = 59.7, *p* < 0.0001, **Figure 1**) between D8 and D15. There was no impact of the cross-fostering treatment on α-diversity indices (GLMM, LogOTU: *F*_1,152_ = 1.3, *P* = 0.26, Evenness: *F*_1,152_ = 1.27, *P* = 0.2, Shannon: *F*_1,152_ = 1.9, *P* = 0.15).

**FIGURE 2:**
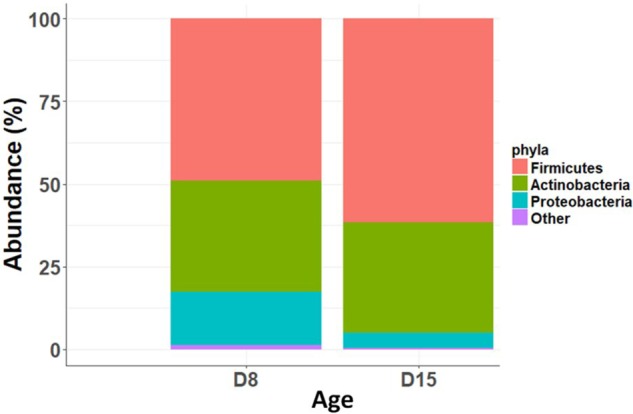
Relative abundance of main bacterial phyla at D8 and D15.

Overall, taxonomic composition was characterized by a predominance of Firmicutes (relative abundance: 46 ± 0.2%) and Actinobacteria (37 ± 0.2%) and a low abundance of Proteobacteria (12 ± 0.17%). There was no significant difference in taxonomic composition between cross-fostered and control nests at D15 (Relative abundance of main phyla: Firmicutes: *t*_1,24_= 0.32, *P* = 0.75; Actinobacteria: *t*_1,24_ = 0.19, *P* = 0.85; Proteobacteria: *t*_1,24_ = -0.47, *P* = 0.64). However, taxonomic composition differed substantially between D8 and D15, with a substantial turnover. At the phylum level, there was a significant decrease in abundance of Proteobacteria (GLMM, *t*_1,141_ = 6.8, *p* < 0.0001 stats) and an increase in Firmicutes (GLMM, *t*_1,141_ = -3.89, *p* < 0.0001) between D8 and D15 (**Figure 2** and barchart with all individuals in Supplementary Figure S1). At lower taxonomic levels, D15 nestlings hosted higher abundances of Bacilli, in particular Lactobacillaceae and Staphylococcaceae and lower abundances of most taxa included in the Proteobacteria and the Bacteroidetes phyla (see Supplementary Figure S2 for linear discriminant analysis (LDA) effect sizes and Supplementary Figure S3 for cladogram).

**FIGURE 3:**
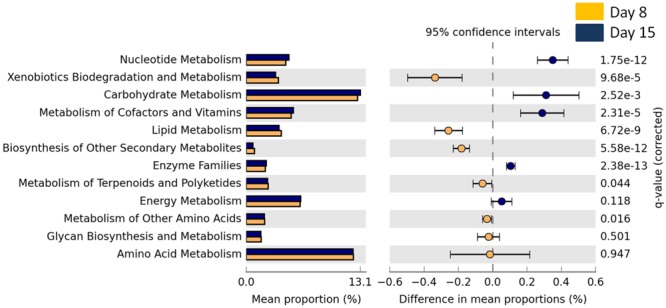
Mean proportion (%) and the difference in the mean proportion (%) of predicted and significantly different (Welch’s *t*-test, Benjamini–Hochberg; *q* < 0.05) KEGG2 metabolic functional inferences of nestling cloacal microbiota at D8 (yellow) and D15 (blue).

The overall abundance of bacteria associated with metabolic functions decreased with age (GLMM, *t*_1,141_ = 9.73, *p* < 0.0001) but there was no effect of cross-fostering (GLMM, *t*_1,153_ = -0.98, *P* = 0.32). We then looked into more specific metabolic functions and found that bacteria associated with xenobiotic degradation and lipid metabolism were significantly more abundant in D8 nestlings whereas those associated with carbohydrate and nucleotide metabolism were more abundant in D15 nestlings (Welch’s *t*-test, corrected *p*-value < 0.0001 for all above features, **Figure 3** and Supplementary Figures S4, S5 for the other significantly different functional features).

### Effect of Cross-Fostering on Microbiota β-Diversity

**FIGURE 4:**
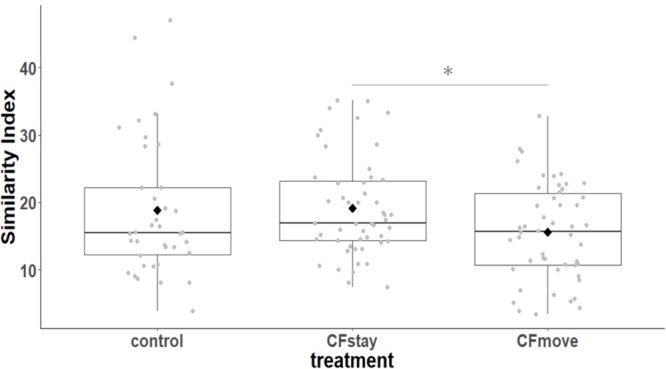
Intra-individual cloacal microbiota similarity (Jaccard distance) between D8 and D15 according to the experimental categories. Median represented by the black line, the mean by the black dot, 25 and 75% quartiles by the lower and upper box and 90% confidence interval by the whiskers. Asterisk refers to statistical differences with *p*-value < 0.05.

**FIGURE 5:**
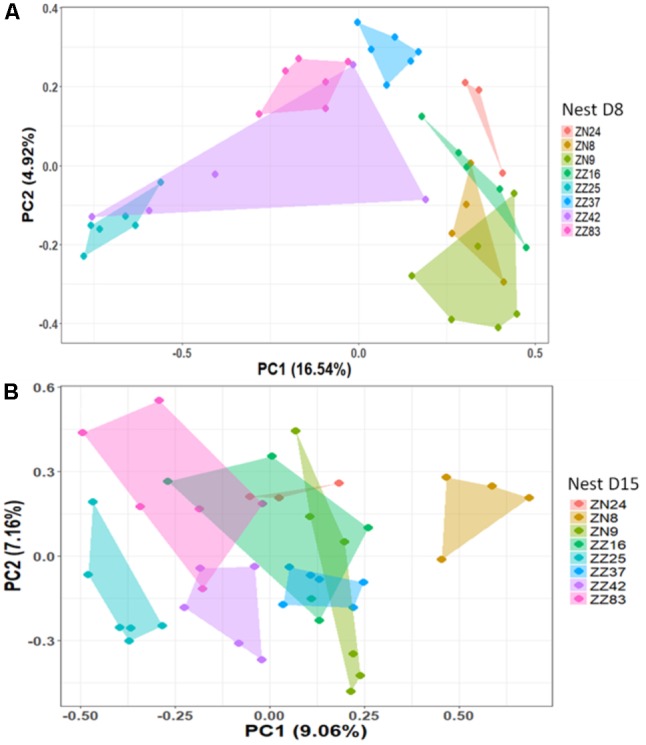
NMDS based on a presence–absence similarity matrix of the cloacal microbiota of the nestlings from the control nests at D8 **(A)** and D15 **(B)**. Each color represents a different nest. Numbers in parenthesis refer to the variance explained by the ordination axis.

**FIGURE 6:**
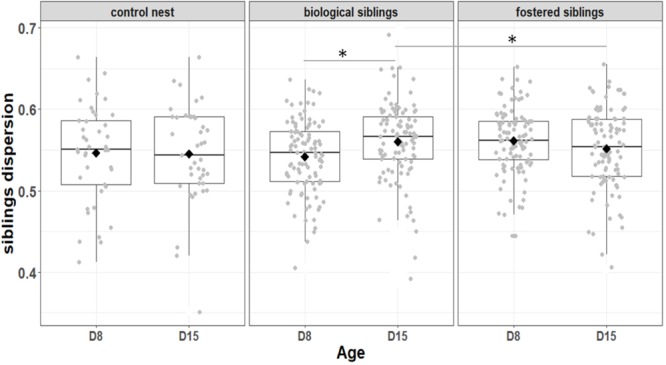
Variation among biological and fostered siblings as indicted by siblings dispersion, that is, the distance of individual nestlings from the centroid of their nest. Median represented by the black line, the mean by the black dot, 25 and 75% quartiles by the lower and upper box and 90% confidence interval by the whiskers. Asterisk refers to statistical differences with *p*-value < 0.05.

**FIGURE 7:**
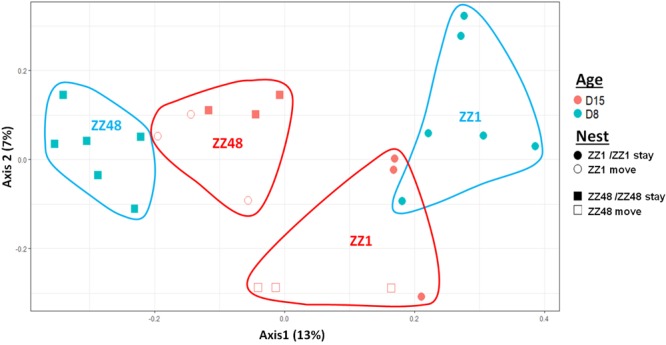
MDS ordination based on a presence–absence similarity matrices of the gut microbiota of the nestlings of a set of cross-fostered nests. Nestlings remaining in their nest of origin are tagged “stay” and those moved to another nest are tagged “move.” Numbers in parenthesis refer to the variance explained by the ordination axis.

We first considered the effect of the experiment on intra-individual microbial similarity between D8 and D15 and found that the microbiota of displaced nestlings (CFmove) changed more than those than remained in the same nest (GLMM, Jaccard distance, *t*_1,82_ = 2.6, *P* = 0.01, **Figure 4**). Similarly, the proportion of D8 OTUs that remained present at D15 was significantly higher in CFstay than in CFmove (26.74 ± 1.68 vs. 21.37 ± 1.70%, GLMM: *F*_1,114_ = 3.63, *P* = 0.03). We then tested the effect of the experiment on inter-individual microbial similarity by examining the correlation between similarity matrices at D8 and D15 at the populational level. This correlation was highest in control birds (Mantel, *R* = 0.37, *P* = 0.001), intermediate in CFstay (Mantel, *R* = 0.13, *P* = 0.003) and low and non-significant in CFmove (Mantel, *R* = 0.07, *P* = 0.06), indicating that the microbiota of displaced nestlings were substantially modified between D8 and D15. PERMANOVA tests revealed an effect of nest identity on inter-individual microbial similarity indicating a higher similarity for nestlings sharing the same nest compared to other nestlings. This intra-nest similarity was strong at D8 (Adonis: all nests, *F*_1,25_ = 1.89, *P* = 0.001), but was lower at D15, although significant, in the CF nests (Adonis: control nest, *F*_1,7_ = 1.78, *P* = 0.001; CF nest, *F*_1,17_ = 1.65, *P* = 0.001). This nest effect on microbial similarity can be visualized in NMDS ordinations (**Figures 5A,B**). In CF nests, the average similarity (Jaccard distance) was significantly higher among foster siblings than separated true siblings at D15 (GLMM, *t*_1,17_ = 2.87, *P* = 0.01), indicating that nest sharing induced a convergence in the microbiota composition of the nestlings. Along the same lines, we considered the degree of clustering of siblings according to age and treatment (distance to centroid, Betadisper procedure). Sibling clustering did not change over time in control nests (GLMM, age, *t*_1,40_ = -0.09, *P* = 0.9), whereas it strongly decreased for separated true siblings (D8 vs. D15: GLMM, *t*_1,100_ = -2.79, *P* = 0.006). At D15, separated true siblings were also less clustered than foster siblings (true vs. foster: GLMM: CF, *t*_1,100_ = -2.84, *P* = 0.005; **Figure 6**). We finally performed an MDS ordination to illustrate individual nestling trajectories in a set of cross-fostered nests (**Figure 7**).

### Gut Microbiota and Nestling Condition

**FIGURE 8:**
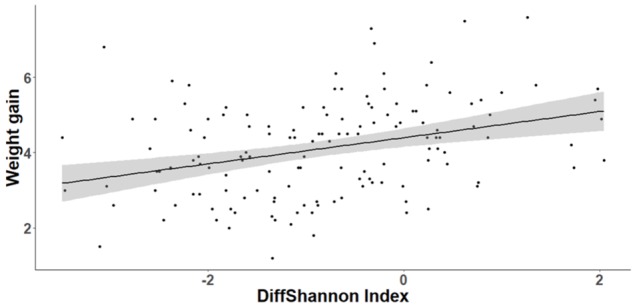
Relationship between individual nestling weight gain and change in cloacal microbiota diversity (Shannon index) between D8 and D15. Gray area around the line refers to the 95% confident interval.

The cross-fostering treatment had no significant effect on either nestling body condition (SMI) at D15 (GLMM, *t*_1,24_ = 0.93, *P* = 0.36) or weight gain between D8 and D15 (GLMM, *t*_1,24_ = 1.01, *P* = 0.32). However, body condition was positively correlated with bacterial richness at D15 (GLMM, *t*_1,112_ = 2.48, *P* = 0.015). Similarly, nestlings that lost fewer bacteria between D8 and D15 gained more weight (GLMM, OTUrichness: *t*_1,112_ = 3.27, *P* = 0.001, Shannon index, *t*_1,112_ = 3.66, *p* < 0.0001, **Figure 8**). The degree of change in microbiota composition was also significantly associated to weight gain (GLMM, *t*_1,112_ = 2.04, *P* = 0.044) with birds with more “stable” cloacal communities (high intra-individual microbiota similarity) gaining significantly more weight. This relationship did not differ among experimental groups (GLMM, similarity^∗^experiment: *t*_1,111_ = 0.53, *P* = 0.6).

## Discussion

In this study, we found that the microbiota of great tit nestlings underwent substantial changes between D8 and D15, with a decrease in diversity and changes in taxonomic and functional composition. Moreover, the partial cross-fostering experiment provided evidence that the nesting environment between D8 and D15 significantly influences microbiota composition, with enhanced changes in nestlings that were fostered to another nest leading to convergence with their foster siblings and a divergence among separated true siblings. Last, we found that higher diversity and stability in microbiota composition were associated with a better host condition.

### Gut Microbiota Dynamics During Nestling Development

Nestlings sampled 8 days after hatching already harbored a diverse gut microbiota with a mean individual richness of 50 OTUs belonging to diverse taxonomic groups. This result shows evidence of a rapid early bacterial colonization of the gut, as has been described in the nestlings of several other wild bird species (Godoy-Vitorino et al., 2008; van Dongen et al., 2013; Kreisinger et al., 2015; Barbosa et al., 2016; Dewar et al., 2017; Grond et al., 2017). Despite this early colonization phase, our results indicate that the microbiota is far from stabilized at D8 with substantial modifications occurring between D8 and D15. We observed a strong overall reduction (28% decrease) in bacterial diversity as nestlings grew older. This was somewhat unexpected as the conventional wisdom is that gut microbiota diversity increases progressively during the first stages of life reaching its apex during adulthood (Palmer et al., 2007; van Dongen et al., 2013; Putignani et al., 2014). Interestingly, Grond et al. (2017) found a similar trend to ours in precocial nestlings, with a decrease in microbiota diversity occurring as early as 3 days after hatching. This decrease during nestling ontogeny could be explained by several non-exclusive mechanisms. The change could be due to (i) “passive” environmental/habitat filtering of the gut on bacterial communities, whereby after initial colonization, only the species best suited to the gut conditions remain and proliferate; (ii) “active” selective recruitment by the host, via the immune system for instance (Stecher and Hardt, 2011), or (iii) by community processes occurring independently of the host, such as competitive exclusion between microbes (Hecht et al., 2016). These three possibilities are in line with the decrease in evenness we observed with age. Moreover nestling hosts undergo several changes (morphological modifications, metabolic changes though diet shifts (Caviedes-Vidal and Karasov, 2001), and maturation of the immune system (Killpack et al., 2013) which may lead to the selection of fewer bacterial species. Whichever the mechanisms involved, our results shed new light on the dynamics of microbial diversity during the ontogeny of avian hosts and indicate that the increase in gut diversity between hatching and adulthood may not be as linear as previously suspected. In fact, it is quite likely that after the decrease we observed during the nestling stage, gut microbiota diversity will increase again after fledging, when the juvenile is exposed to a wide range of novel environments and food sources (cf. Godoy-Vitorino et al., 2010). Further studies examining the gut microbiota at later stages of development (fledging, first-year birds, etc.) are needed to better understand the overall ontogenetic dynamics of gut microbiota diversity in birds.

Alongside this decrease in diversity with age, we observed substantial changes in the taxonomic composition, namely a significant increase in the relative abundance of Firmicutes and a decrease in Proteobacteria between 8 and 15 days. Interestingly, similar taxonomic shifts have been observed in developing nestlings of other bird species, such as hoatzin (Godoy-Vitorino et al., 2008), little penguin (Dewar et al., 2017), and arctic shorebirds (Grond et al., 2017). The fact that the same compositional shifts are found in species with such contrasting ecologies could possibly indicate that this is a widespread pattern in young birds, which could be associated to particular ontogenetic functional shifts. For instance, Firmicutes are known to be involved in the fermentation of organic molecules and have been positively associated to weight gain and fat storage in both avian and mammalian models (Turnbaugh et al., 2006). In particular, the increase of Lactobacillaceae (a family belong to the Firmicutes phylum), which are also significantly more predominant in 15 days nestlings in our study (Supplementary Figure S3), has been found to be associated with higher weight gain in broiler chicks and ducklings (Angelakis and Raoult, 2010). In this context and given the apparent widespread occurrence of this gut community shift in young birds, it is plausible that the increase in Firmicutes and *Lactobacillus* spp. in particular, may be adaptive by facilitating weight gain during this period of rapid nestling development (Monrós Juan et al., 2003; Rodriguez et al., 2016). Because Proteobacteria are characteristic environmental bacteria (Grond et al., 2017) and are generally associated with higher risk of gut dysbiosis (Shin et al., 2015), the decrease we observe with age may comprise further evidence of selective recruitment of gut communities by the host.

The examination of the functional profile of nestling gut metagenomes inferred using PICRUSt may provide additional insight in functional shifts associated to nestling growth. The higher metabolic potential to detoxify xenobiotics in the gut microbiota of younger compared to older nestlings, for instance, could corroborate the notion that the gut is exposed to many novels compounds in the early days after hatching. The shift in metabolic profiles from more abundant lipid metabolic features in 8-day-old nestling toward more abundant carbohydrate or nucleotide metabolic features in 15-day-old nestlings, on the other hand, could reflect dietary shifts throughout nestling development (Wiebe and Slagsvold, 2014; Wesołowski and Neubauer, 2017). The higher level of lipid metabolic functions in 8-day-old nestlings could be explained by nestling nutrition in the first days after hatching. Until parental food is supplied, residual yolk found in the abdominal cavity (20% of hatchling weight in poultry, Sklan and Noy, 2000) is the only nutrient source for hatchlings. This lipid-rich residual yolk (e.g., Toledo et al., 2016 for yolk composition in great tits) comprises a substantial source of nutrients for several days after hatching (Panda et al., 2015) and could select for bacteria taxa involved in lipid metabolism in the early stages of nestling ontogeny.

### Influence of Nest Environment as Shown by Cross-Fostering

Our results showed a strong nest effect on microbiota composition, with higher similarity for nestlings sharing the same nest compared to other nestlings at both D8 and D15. This finding corroborates that of previous studies on altricial birds (Lucas and Heeb, 2005; Ruiz-Rodriguez et al., 2009; Kreisinger et al., 2017), highlighting the major role of the rearing environment in shaping the gut microbiota of nestlings. However, this nest effect confounds several factors such as nestling genetic makeup, parental effects or characteristics of the nest itself. In this context, the cross-fostering experiment gives us an opportunity to partially disentangle these confounding factors and better understand the contribution of the later rearing environment in mediating the changes observed between D8 and D15.

The fostering treatment induced significant changes in the microbiota composition of fostered nestlings compared to controls. In addition, we found that fostered nestlings at D15 were more similar to their foster siblings than to their true siblings, implying significant convergence of the gut microbiota of nestlings developing in the same nest. Thus, although age is the main force driving microbial change between D8 and D15, the rearing environment still significantly contributes to shaping the gut microbiota at an intermediate stage of nestling development. Our results further indicate that the factors explaining intra-nest similarity at D8, be they genetic, parental or common environment, are largely overrun by the influence of the rearing environment between D8 and D15.

The effect of the D8–D15 rearing environment can be explained by several factors. First, changes could be attributed to parental effects, as our cross-foster experiment effectively changed the parents of the fostered nestlings. A study on barn swallows (*H. rustica*) by Kreisinger et al. (2017) found that mothers and nestlings shared similar gut communities, indicating a possible vertical transmission through feeding. Such vertical transmission through feeding seems less likely in our species, however, as preys are fed directly without parental ingestion or regurgitation as is the case in swallows. An indirect parental effect is still possible through variation in feeding investment or prey type for example (Pagani-Núñez et al., 2015; Mathot et al., 2017), given the importance of diet in shaping the gut microbiota (Wu et al., 2011) A second possible mechanism explaining the nest effect is horizontal transmission of bacteria between nestlings, leading to homogenization of their gut microbiota. Indeed the social environment has been found to shape the gut microbiota (Tung et al., 2015). Here, our cross-fostering design allows us to differentiate the influence of nestmates relative to that of the nesting environment *per se*. The change in microbiota was lowest in control nestlings, intermediate in nestlings that received new foster siblings, and highest in siblings displaced to a new nest. This provides evidence that, although the social environment does play a role, its influence is lower than that of the nesting environment *per se.* This brings us to the third possible factor contributing to the nest effect, which is the influence of the nest composition on the nestling gut microbiota. Variations in nest material composition, size, and weight have been found within great tits populations (Álvarez et al., 2013; Lambrechts et al., 2017), which result in differences in the bacterial communities associated to the nests (Goodenough and Stallwood, 2010; Brandl et al., 2014) or nest boxes (Goodenough and Stallwood, 2011). These bacteria in turn are then likely to colonize the skin (Gonzalez-Braojos et al., 2015), feathers (Leclaire et al., 2015), and gut (through preening for instance, see Kulkarni and Heeb, 2007 for plumage-gut transmission) of the birds (Goodenough et al., 2017; van Veelen et al., 2017). These three main factors (parental effects, siblings, nest environment) are not exclusive and may all contribute to various extents to the rearing environment influence we observed in this study. Further analyses comparing the gut microbiota of nestlings with that of their parents (both foster and biological) as well as the bacterial communities in the nest material could help us better understand the relative contribution of each factor.

### Implications for Host Fitness

Our study shows several interesting relationships between gut microbiota characteristics and nestling body condition, which has hardly been studied in wild birds. First, nestlings with higher microbiota diversity had a higher relative body mass at D15. One of the explanations of this positive effect, can be that more diverse gut communities are more resistant to pathogens invasions, and in general are more stable and resilient following perturbations (Buffie and Pamer, 2013). A lower microbiota diversity is usually considered to be detrimental to hosts (Le Chatelier et al., 2013) because it entails a loss of essential functions leading to reduced nutrient assimilation or immunodeficiency, for instance. Second, nestlings that had the most stable microbiota both in terms of diversity (lowest decrease) and composition (high intra-individual similarity over time) showed the highest weight gain between D8 and D15. While the causality of this correlation cannot be proven with the present data, major modifications in the microbiota community can lead to dysbioses that can induce deleterious effect for the host (Logan et al., 2016), which could explain the lower weight gain in nestlings with more inconsistent microbiota. Body condition is an important factor for bird fitness, especially for nestling survival after fledging (Monrós Juan et al., 2003; Rodriguez et al., 2016) highlighting the role of the gut microbiota and the possible consequences of disturbed microbiota on nestling fitness.

Overall, our study shows evidence that the gut microbiota undergoes multiple changes in diversity, taxonomic, and functional composition within a short timespan during nestling development. It also provides evidence that nesting environment continues to shape microbiota during the later stages of nestling development. Lastly, the microbiota changes incurred during this period may have implications for nestling body condition which can lead to long-term consequences for host fitness.

## Author Contributions

AT, JW, and EM: design of the study and data analysis. AT: field sampling. AT and JW: laboratory analysis. JW, LL, and EM: financial funding. AT, JW, LL, and EM: manuscript drafting. All authors provided helpful comments and recommendations and approved the final version of the manuscript.

## Conflict of Interest Statement

The authors declare that the research was conducted in the absence of any commercial or financial relationships that could be construed as a potential conflict of interest.

## Footnotes

1 http://huttenhower.sph.harvard.edu/galaxy/
