# Supplementary material for: Dynamics of Gut Microbiota Diversity During the Early Development of an Avian Host: Evidence From a Cross-Foster Experiment
Source: Front Microbiol. 2018 Jul 9;9:1524. doi: 10.3389/fmicb.2018.01524 (PMC6046450; doi:10.3389/fmicb.2018.01524)
Supplement: Supplementary file 1 [file Data_Sheet_1.docx]

Supplementary Material

**Dynamics of gut microbiota diversity during the early development of an avian host: evidence from a cross-foster experiment**

**Aimeric Teyssier^*^, Luc Lens, Erik Matthysen and Joël White**

*corresponding author: aimeric.teyssier@uantwerpen.be

**
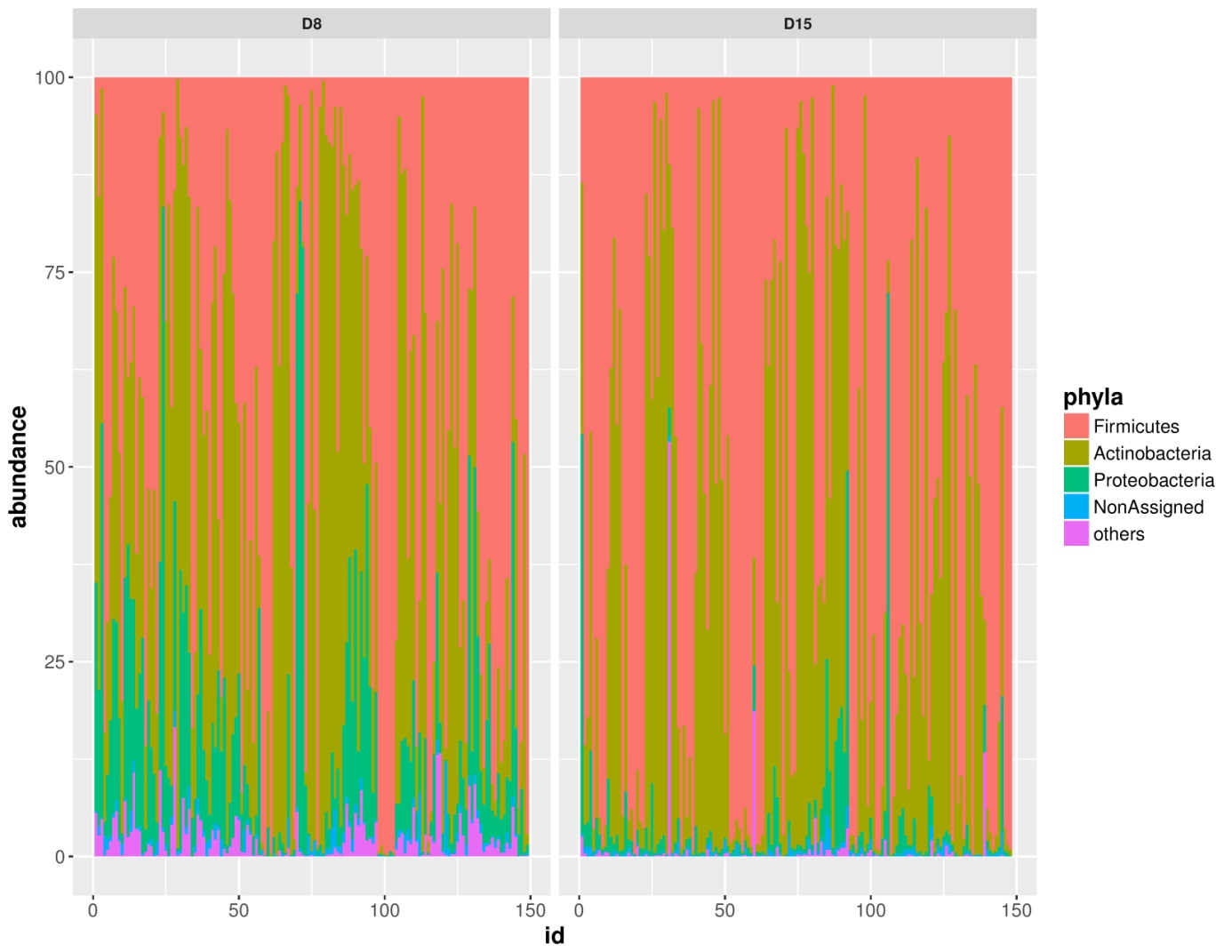
**

**Supplementary Figure 1:** Phylum abundance bar chart of the cloacal microbiota of all the individuals sampled according to the age of the nestlings


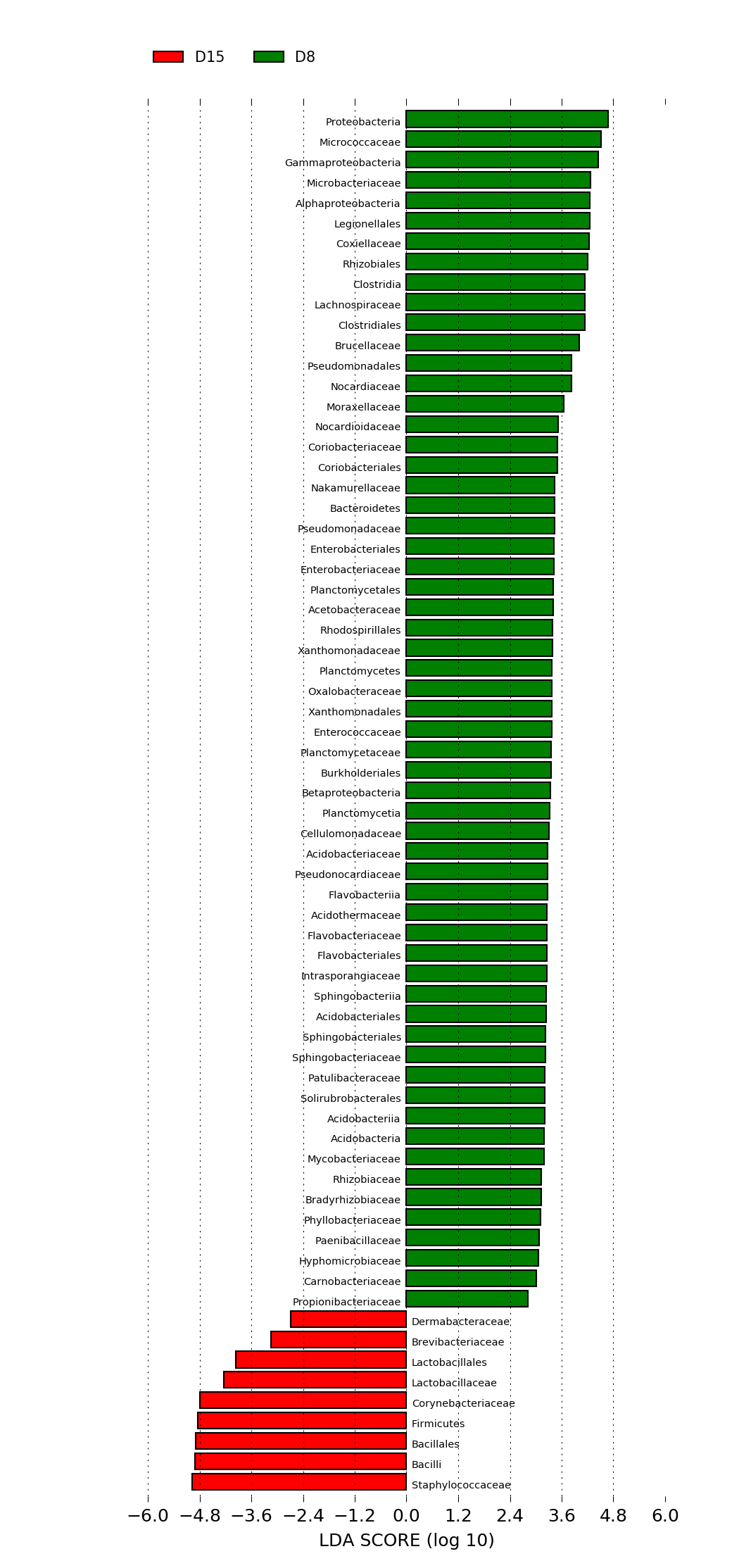


**Supplementary Figure 2**: Linear Discriminant Analysis (LDA) of the taxa identified in the great tit nestling gut microbiota. Green and red colors represent taxa with significantly higher abundance at D8 or at D15 respectively.


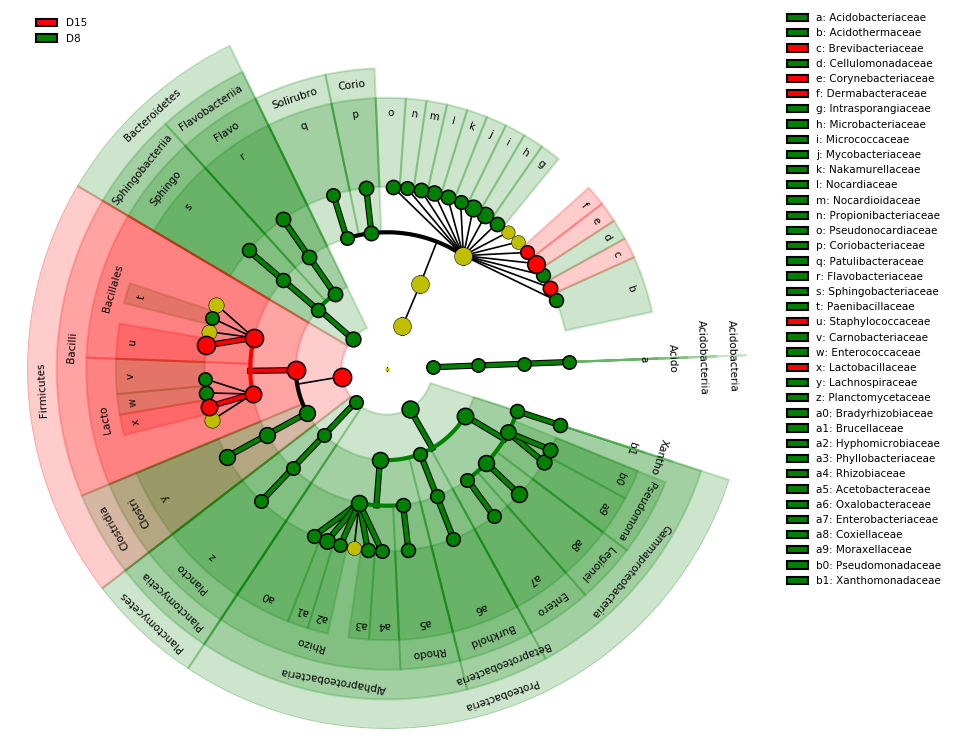


**Supplementary Figure 3**: cladogram of the microbiota composition at D8 and D15. From inner circle to outside: phylum, class, order and family. Green and red colors represent taxa with significantly higher abundance at D8 or at D15 respectively. Yellow color represents taxa with no significant differences between D8 and D15.


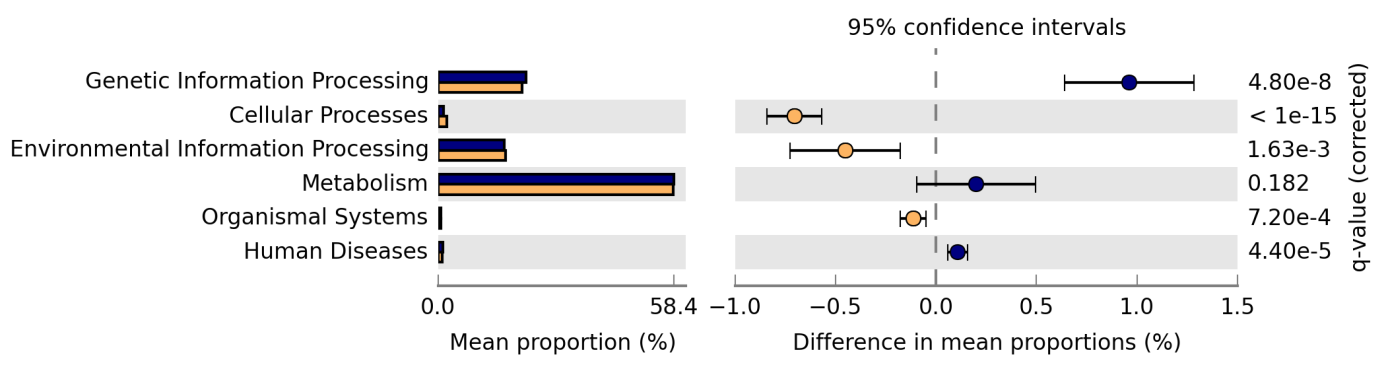


**Supplementary Figure 4**: Mean proportion (%) and the difference in the mean proportion (%) of predicted and significantly different (Welch's *t*-test, Benjamini-Hochberg; *q* < 0.05) functional inferences of nestling gut microbiota at D8 (orange) and D15 (bleu).


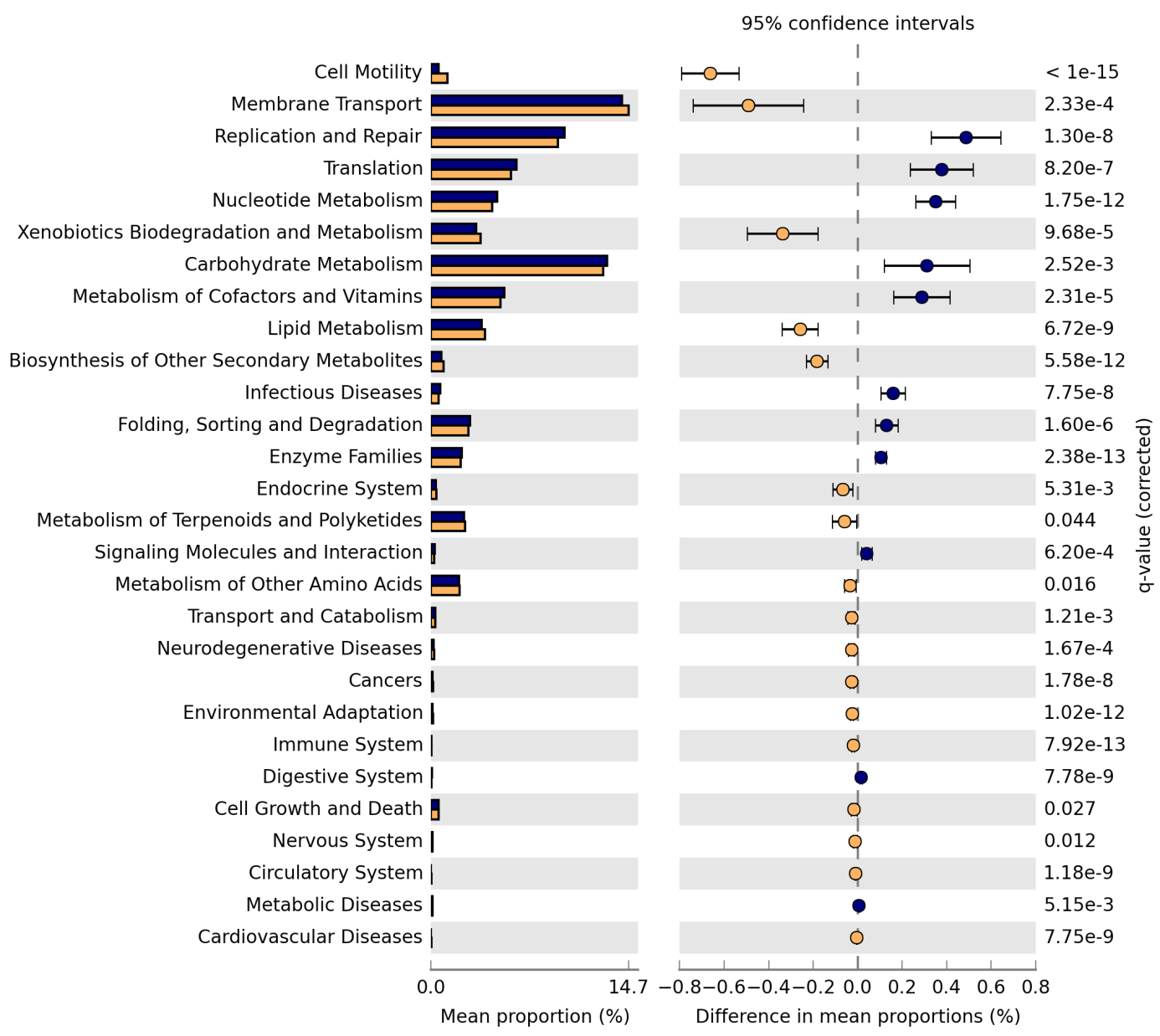


**Supplementary Figure 5**: Mean proportion (%) and the difference in the mean proportion (%) of predicted and significantly different (Welch's *t*-test, Benjamini-Hochberg; *q* < 0.05) KEGG 2 functional inferences of nestling gut microbiota at D8 (orange) and D15 (blue).

**Supplementary data sheet 2**: taxonomic assignation and abundance of the OTUs characterized as contaminants.

**Supplementary data sheet 3**: detail of the OTUs that managed to map (success) or not (fail) the greengenes reference.
